# A qualitative study of infection prevention and control practices in the maternal units of two Ghanaian hospitals

**DOI:** 10.1186/s13756-023-01330-z

**Published:** 2023-11-13

**Authors:** Gifty Sunkwa-Mills, Kodjo Senah, Mette Breinholdt, Matilda Aberese-Ako, Britt Pinkowski Tersbøl

**Affiliations:** 1 Ghana Health Service, Awutu Senya East Municipal, Kasoa, Central Region Ghana; 2 Global Health Section, Department of Public Health, University of Copenhagen, Copenhagen, Denmark; 3 Department of Sociology, University of Ghana, Legon, Accra Ghana; 4 Hermes Interaktion, Silkeborg, Denmark; 5 Institute of Health Research, University of Health and Allied Sciences, Ho, Ghana

**Keywords:** Healthcare-associated Infections, Low- and middle-income countries, Healthcare providers, Postnatal phase, Women, Ghana, Infection prevention and control

## Abstract

**Introduction:**

Healthcare-associated infections (HAIs) remain a common challenge in healthcare delivery, with a significant burden in low- and middle-income countries. Preventing HAIs has gained enormous attention from policy makers and healthcare managers and providers, especially in resource-limited settings. Despite policies to enforce infection prevention and control (IPC) measures to prevent HAIs, IPC compliance remains a challenge in hospital settings. In this study, we explore the experiences of healthcare providers and women in the post-natal phase and investigate factors influencing IPC practices in two hospitals in Ghana.

**Methods:**

The study used a qualitative approach involving semi-structured interviews, focus group discussions, and observations among healthcare providers and women in the postnatal phase in two maternity units from January 2019 to June 2019. Interviews were recorded and transcribed verbatim for thematic analysis. The data sets were uploaded into the qualitative software NVivo 12 to facilitate coding and analysis.

**Findings:**

Healthcare providers were driven by the responsibility to provide medical care for their patients and at the same time, protect themselves from infections. IPC facilitators include leadership commitment and support, IPC training and education. Women were informed about IPC in educational talks during antenatal care visits, and their practices were also shaped by their background and their communities. IPC barriers include the poor documentation or ‘invisibility’ of HAIs, low prioritization of IPC tasks, lack of clear IPC goals and resources, discretionary use of guidelines, and communication-related challenges. The findings demonstrate the need for relevant power holders to position themselves as key drivers of IPC and develop clear goals for IPC. Hospital managers need to take up the responsibility of providing the needed resources and leadership support to facilitate IPC. Patient engagement should be more strategic both within the hospital and at the community level.

**Supplementary Information:**

The online version contains supplementary material available at 10.1186/s13756-023-01330-z.

## Introduction

Healthcare-associated infections (HAIs) are among the most common patient safety risks in healthcare worldwide [1, 2]. HAIs are known to contribute to significant morbidity, mortality, and financial losses in healthcare systems [3, 4]. The risk of maternal deaths from HAIs has been known for a long time and remains an issue of global concern [5]. The possibilities of prevention of HAIs, particularly puerperal sepsis, through sanitary practices and birth surroundings, are also known. Infection prevention and control practices (IPC) have been known to contribute greatly to HAI prevention and improvement in the quality of care patients receive [2].

There is a global call to take steps to ensure the prioritization of high-quality maternal health care for women and girls [6, 7]. The prevalence of HAIs remains a threat to efforts to promote quality care that is effective and safe for patients [8]. Although IPC is important in HAI prevention, IPC practices are adopted to varying extents in hospital wards. In 2016, there was a call to action in the Lancet to urgently prioritize improvements in the quality of care during pregnancy, delivery, postpartum, and beyond [7].

The Ghana Health Service (GHS) and other agencies of the Ministry of Health (MOH) are paying attention to issues of quality service delivery, having put in place some strategies to improve care [9]. Despite efforts by GHS and the MOH to improve the quality of care, there are health system challenges in the implementation of IPC interventions [10–13]. The COVID-19 pandemic was a further reminder of the risks that healthcare providers face from exposure to HAIs in hospital wards [14]. There is a need for more research to explore the experiences of improving quality care delivery in hospitals.

IPC involves different players in the hospital, and everyone has a role to play based on their experience, knowledge, and opportunities to act and engage within the complex healthcare system [15–17]. While hospital managers have a critical role to play in guiding IPC policy implementation and resource distribution, frontline health workers also play practical roles in shaping how policies are implemented [18–20].

In low- and middle-income countries (LMICs) including Ghana, there is sparse literature on the lived experiences and interactions between hospital managers, healthcare providers and clients in the complex ward environment, and how this influences IPC practices. In this paper, we investigated the perceptions and practices of IPC among healthcare providers and women in the postnatal phase and explored contextual factors influencing IPC compliance and care delivery in the maternal wards of two hospitals in Ghana.

We apply VeneKlasen and Miller’s concept of power to interpret and discuss the findings [21]. In this concept, power is defined as “an individual, collective, and political force that can either undermine or empower citizens and their organizations … its expressions and forms can range from domination and resistance to collaboration and transformation.” Power emanates from political, financial and bureaucratic sources, and also resides in professional status, gender norms, knowledge and technical expertise. Dimensions of power are categorized as ‘power over’ (political, hierarchical, etc. authority), ‘power with’ (individual agency to resist internalizing discrimination), ‘power to’(potential to shape one’s life and world)” and ‘power within’ (sense of self-worth and self-knowledge). These forms of power manifest in various ways in interactions between healthcare providers and their supervisors and also between healthcare providers and their patients [21, 22].

## Methods

### Study setting

Ghana has 162 district hospitals, 10 regional-level hospitals, and five teaching hospitals (tertiary-level) in the public health sector. District hospitals form the first referral point for health centers and polyclinics, regional hospitals form the secondary-level referral point, and teaching hospitals provide tertiary-level care [23, 24].

The study was conducted in the maternal units of a tertiary-level hospital (TH) and a secondary-level hospital (SH) in southern Ghana. The study sites, dubbed TH and SH, provide outpatient and inpatient services to populations of 5 million and 3 million, respectively. TH has a 2,000-bed capacity, while SH has a 400-bed capacity. HAI prevalence is 10.2% in TH and 10.0% in SH, both above the national average of 8.2% [25]. The study was conducted within the context of a larger hospital-based project investigating healthcare-associated infections in Ghana, the HAI-Ghana project.

### Study design

A qualitative ethnographic approach was used [26], employing in-depth interviews, focus group discussions (FGDs), and observations.

Healthcare providers of various cadres were purposively selected [27] to participate in the study. The selection of participants was based on a representative purposive sampling technique (maximum variation) [28–30]. We selected staff in each of the main cadres (prescribers, nursing staff, auxiliary staff) working within the hospitals. Health providers who had worked in the wards for 6 months or more were selected because they provide day-to-day care for women in the pre-and postnatal phase, and interact with the mothers. This position offers them rich experience and knowledge in the area of study. Senior management members with over 5 years of experience were included to share their experiences from an administrative perspective and to increase the wealth of experience shared. Health managers (senior medical officers and physician assistants in charge), ward managers (senior nurses/midwives) and IPC coordinators were purposively selected because, as managers in the facility and wards they are responsible for making key decisions involving the implementation of IPC policies in the study facilities. Consequently, the study was able to access this rich data source. We excluded staff working at the outpatient departments and staff on study leave or transfer during the study.

Women in the postpartum period who were on admission to the wards 48 h or more during the study period and who were in stable condition were invited to participate in the study. Selection was by convenience sampling, taking into consideration their availability, and willingness to participate and share their experiences with researchers [28]. These women were selected because they were both mothers and carers of babies and over the period had rich experience in the subject matter and so could help the team to achieve the objectives of the study. The women were approached face to face and invited to participate in in-depth interviews or FGDs after the study had been explained to them. None of the invited participants refused to participate.

### Data collection

Data were gathered from the study sites between January 2019 and June 2019. Selected participants were informed about the study’s objective and data collection processes. The research team was made up of the first author, GSM, a female medical doctor and a Ph.D. student with qualitative research experience, and two research assistants (RAs) who have degrees in health-related fields and who could speak the Twi language, which is commonly spoken in the study areas. In the selection of research assistants, a crucial consideration was their ability to speak, read and write in the Twi language. The first author, GSM, trained the research assistants in data collection and recording field notes and observations for qualitative research.

Twenty health providers (HPs) participated in the in-depth interviews. Interviews were conducted using a semi-structured interview guide (Appendix 1) that was developed and pilot-tested by the research team. The design of our semi-structured interview guide was informed by the objectives of our study and relevant literature including the WHO’s “Guidelines on Core Components of Infection Prevention and Control Programmes at the National and Acute Health Care Facility Level” [31, 32]. Our thematic areas were guided by four core components of the Infection Prevention and Control (IPC) Assessment Framework (IPCAF): IPC program; IPC guidelines; IPC education and training; and HAI surveillance [33].

Interviews were performed at a location of the participant’s choice, mostly a quiet side room in the hospital. A total of 12 women in the postnatal phase participated in the in-depth interviews, and 44 women in the postnatal phase participated in 6 FGDs, with 6–8 women per group. Interviews were conducted face-to-face, and participants were interviewed alone in private rooms in the hospital. Interviews lasted between 30 and 60 min, and FGDs lasted 60–90 min. Women were asked to share their experiences in the hospital and the maternity wards. They were also asked questions about their experience with puerperal sepsis. Probing was done when necessary to clarify the responses given.

Some interviews were conducted in Twi upon the request of mothers. The first author moderated the FGDs, while two research assistants, took observational notes. The interviews were audio-recorded and transcribed verbatim; those conducted in Twi were translated into English by the research assistants. Before the study’s commencement, researchers did not establish a relationship with the participants. However, the objectives of the research were explained to participants, and they were allowed to ask any clarifying questions. It was also made clear that participation was voluntary.

The first author conducted observations for up to 8 weeks in each study site using an observation guide. Over the period of the research over 140 h of observation were conducted over day and night shifts at the study sites. Field notes from observations were documented to provide further insights into the study context [34]. Participants were offered snacks as refreshments after the interviews.

### Data management and analysis

Transcripts and notes were kept confidential in password-protected files. Transcripts were read to develop a coding structure reflecting issues arising from the data. After becoming familiar with the data, the first author generated initial codes across the data set. Coded data were grouped, and themes were identified [35]. Coding of the transcripts continued using NVivo 12, and commonly occurring themes and subthemes were identified. Coding, thematic analysis, and data collection continued until saturation was reached and no new information was obtained [30, 34, 36].

Strategies were employed to ensure the trustworthiness of the findings, such as pretesting interview guides to ensure rigor, and checking the data for accuracy and completeness [37, 38]. In presenting our findings, quotes from participants are identified by codes for confidentiality. We used the COREQ (Consolidated Criteria for Reporting Qualitative Research) checklist to report our findings.

The position of the first author as a medical doctor and a public health specialist was reflected upon during data collection and analysis. This is to reflect on how her knowledge and experience of the hospital context might influence how data is collected and analyzed. The research was supervised by the principal supervisor (last author, BPT) and a co-supervisor (second author, KS), both of whom have many years of qualitative research experience.

## Results

The demographic characteristics of healthcare providers are provided in Table 1.

**Table 1:** Characteristics of healthcare providers who participated in the study (n = 20)

Demographic characteristics	Number	(%)
Age		
20–30	8	40
30–39	10	50
> 40	2	20
Staff cadre		
Manager	2	10
Doctor/Physician Assistant	5	25
IPC Coordinator	2	10
Nurse/Midwife	7	35
Other (Cleaner, Health Assistant)	4	20
Years in current position		
0–5	10	50
6–10	8	40
> 10	2	10
Location/Service Level		
Tertiary (TH)	10	50
Regional (SH)	10	50
Gender		
Male	8	40
Female	12	60

The demographic characteristics of women who participated in the study are described in Table 2.

**Table 2:** Characteristics of women who participated in the study

Demographic characteristics	Number		
	FGDS	IDIs	Observations
Age			
15–19	4	1	5
20–29	22	5	27
30–39	14	4	18
40–49	4	2	6
Marital Status			
Single/other	14	4	18
Married	30	8	38
Educational Level			
None	6	1	7
Primary	18	4	22
Secondary	12	4	16
Tertiary	6	3	9
Postgraduate	2	0	2

### The hospital context

At 6:30 a.m. on a Monday, the cleaner, Alhassan, was already at work, mopping the long corridors that opened into the patient wards. His mop bucket was half-filled with a lathered solution - water mixed with soap, chlorine and disinfectant - that left a fragrance on the corridors. He mopped from the main entrance right through to the end of the ward, and adjacent corridor. Even while he cleaned, a few visitors tried to walk past him to see their relatives. He complained that they would dirty the floors, and then the doctors and nurses would think he had not worked.

While he was mopping, the matron-in-charge arrived on the ward, quickly noticed and commended the good work he was doing. This energized him. When he mentioned to the matron that the visitors were ‘disturbing’ him, she immediately ordered the security man to shut the doors and restrict further entry by visitors.

Once the matron sat down at the nurses’ desk and settled in, a few nurses gathered around her- including those from the night shift and those who had arrived from home for the morning shift. The team then went through the routines of ‘handing over’ and ‘taking up’, with the night staff giving an account of the night shift, admissions and discharges, and moving around from bed to bed to show whose condition was stable or unstable and who needed further care. Once this was done, the matron-in-charge instructed the junior nurses to straighten up the patients’ beds and check their vitals, while a senior nurse supervised the setting up of the Rounds Trolley and equipment in preparation for the doctors’ arrival for the ward rounds.

This scene above is one of many observations of a typical morning in the maternity wards. Various actors coordinate to facilitate the provision of care for women in the maternity wards.

Both hospitals have a medical director who is the head of the hospital management team. The core hospital management team comprises the clinical coordinator, administrator, accountant, pharmacist, and director of nursing services. The roles of the management team include planning, organizing, coordinating, budgeting, innovation, representing the organization, etc. The management teams have regular meetings to discuss administrative and clinical issues and make relevant decisions on service delivery and the running of the hospital.

The maternity wards have a ward manager (senior nurse) or ‘matron-in-charge’, who oversees the day-to-day administration of the ward and is directly responsible for supervising nurses and auxiliary staff in the wards. There is also an overall head of the unit who is a Specialist Obstetrician and who supervises other medical doctors on the unit. Medical and nursing students are often found on the wards for short rotations or internships. Healthcare assistants (with some basic training in nursing care) and “orderlies” (cleaners) perform various roles on the wards. Within this context, we identified several facilitators and barriers to healthcare delivery and IPC compliance.

### Facilitators and barriers of IPC

We identified four themes denoting IPC facilitators: Leadership commitment and support, Perception of IPC as a form of professional care for patients, Training and education for IPC, and IPC as a form of Self-care. Five thematic areas represent IPC barriers, comprising, the ‘invisibility’ of HAIs, low prioritization of IPC by managers, Lack of resources and sufficient goals for IPC, discretionary use of protocols, and communication-related challenges.

### Facilitators of IPC compliance

#### Leadership commitment and support towards infection prevention and control

When asked about IPC policies and implementation, hospital managers emphasized their commitment to improving IPC on the wards. A manager said:

Well, infection prevention is a priority, and we see it as an obligation, so we provide the necessary funds. Because if you don’t, it’s either the patients are being infected or the staff are being infected, and either way, this is increased cost. So why not do the prevention rather than the cure? … apart from the inconvenience, it causes more morbidity to the patient, and the length of hospital stay is increased and all that… also, it affects the image of the hospital in terms of quality care. (IDI-1 M, Manager)

Apart from the management team, there were other operational teams in the hospitals to whom various tasks were assigned. Some actions by management were identified to be supportive of IPC. These included the formation of IPC committees in TH and Quality Assurance committees in SH to identify gaps in IPC and provide support to improve IPC and the delivery of quality care. In TH, IPC focal persons, also known as IPC champions, were appointed among the healthcare providers as local ambassadors for IPC. Part of their responsibility was to drive the implementation of IPC protocols on the wards. In SH, the Quality Assurance team is responsible for overseeing quality care delivery including IPC policy implementation in the hospital. This team was headed by a Senior nurse who is also in charge of IPC. She mentioned that she was responsible for coordinating IPC activities and conceptualizing innovative ways to improve IPC on the wards.

During observations and interactions with the various clinical teams, doctors and nurses discussed the shared value of safe practice and quality care and emphasized the importance of teamwork. Doctors are in charge of leading ward rounds, and other health providers participate in the ward rounds. Patient conditions and management plans are discussed during the rounds. Doctors delegate tasks to other team members based on the workload and team dynamics, and as they deem appropriate.

Nurses were careful to dedicate time during their shifts to ‘handing over’ and ‘taking up’.

A Matron-in-charge narrated:

I have to schedule the work for everyone to work. We have six suites, so I put one midwife in each suite, and if there are rotation midwives or nurses, I add them up…then I send someone to the theatre to receive the babies there. Then … we have to do admissions and discharges as well. …If there is any difficult delivery, then I have to be called … sometimes I have to call on a doctor to come and intervene. (IDI-3 F, Midwife).

Nursing staff took care of ward logistics and administrative issues such as documentation of admission or discharge of patients and attending meetings. Documentation was done by the nurse-in-charge at the beginning and end of every shift, to show work done during the shift, and patient numbers and conditions. One of the nurses disclosed that doing this right signifies the power or authority of the matron-in-charge and reflects responsibility toward her role.

A midwife stated:

OK, when we come in the morning… you take up from the previous shift. After taking up, you go through the ward to give medications. Then, maybe, we share our roles in the various suites … (IDI-1 F, Midwife).

Another nurse stated the following:

… after taking up, we pack our delivery instruments for sterilization… then we go to the laundry for bedsheets, delivery towels, and theatre gowns. Then, after that, if anyone is in labour, you monitor them. (IDI-2 F, Midwife).

The management of patient conditions was regarded as essential by healthcare providers. Core clinical tasks are more closely associated with expertise and therefore more gratifying as expressed by *IDI-3 F* (midwife), who proudly described how her colleagues draw on her expertise or that of the doctors “if there is any difficult delivery”.

Administrative tasks were often associated with status. In SH, a manager explained the various administrative tasks he must perform due to his status as a director. When asked to give the highlights of a typical workday, he responded:

On Mondays, we will have an Obs and Gynae Quality Assurance meeting from 7:30 am to 9:30 am, then a management meeting up to about 11:30. … Then I go around… just to look at various places in the hospital and how things are going. Tuesday is a typical clinical workday for me, so ward rounds from 7:30 am to about 10:30, then I go to the outpatient clinic, which will take almost the rest of the day… Wednesday is an Obs day, I may have a few surgeries… after that, I’ll come and do office work… Thursday is basically administrative stuff, so again, I work in the office; after office work, I go around. Then Friday is basically a theatre day for me… So, from the theatre, I’ll come back to the office. On the weekend if I’m on duty… I take it up. In terms of administrative work, I sit at the office and do some administrative work… signing stuff, and after that, meetings. So, I schedule most of the administrative meetings for Wednesday or Thursday when I do little clinical work.

Time was also dedicated to meetings involving senior staff who have the responsibility for decision-making on the wards. Due to the authority and power held by managers in these roles, their administrative roles are respected, and their instructions are taken seriously by other staff. However, the long hours spent at these meetings and on documentation take time away from rendering care or supervising care delivery. Moreover, the focus of these meetings and documentation is mainly biomedical, and IPC may be evaded.

#### Healthcare providers’ responsibility to render professional care to patients

Resident doctors, medical officers, and interns stayed behind to manage the wards after the morning ward rounds. They reviewed patients’ folders, performed any tasks assigned by the consultants during ward rounds and took care of any new admissions and emergencies arising. Healthcare providers dedicated efforts to clinical care as there are potentially great repercussions if a healthcare provider slacks or makes mistakes. On ward rounds, senior doctors sternly reprimanded other staff when clinical protocols were not followed, or medications were missed or omitted. A nurse spoke about the need to minimize the spread of infections from shared medical devices, by cleaning them well after they have been used on any patient. We observed efforts to clean thermometers with ‘methylated spirit’ after every use.

Clinical emergencies were attended to promptly. It was indicated that sometimes a doctor could be called while conducting ward rounds to attend an emergency in the theatre. A few health providers described the different ways in which they react when handling emergencies. A midwife mentioned how the use of PPEs may sometimes be compromised when handling such cases.

Normally, we wear two gloves when delivering. You use the first one to conduct the delivery then you change it. But sometimes there could be an emergency case after you have already delivered the baby… If the baby is not crying, you have to resuscitate the baby. At times, you even forget to remove the first glove (IDI-2 F, Midwife).

Two midwives indicated that they would go ahead and receive an emergency birth even if they were not adequately gowned or gloved, as it involved saving a newborn or a mother’s life.

A healthcare assistant described scenarios where she exercised fewer precautions, or precautions were not taken even when it was indicated, for example, when a patient was an acquaintance.

For patients with whom staff were acquainted e.g., other staff members or influential people, a manager described that “we sometimes go the extra mile for them”, but quickly added: “There is only so much you can go above your regular standards or levels of care”. In making a case to advocate for managerial and political commitment to improve the infrastructure and equipment within the hospital, he added: “Get people in power to use the facilities. If it doesn’t touch their skin, they don’t feel it much”.

Orderlies like Alhassan described the importance of their work in ensuring that the ward was clean so that healthcare providers could do their work without any distractions. Orderlies were assigned some other tasks, such as laundry and disinfection of equipment, from time to time.

#### Education and training for IPC

Management members informed us during the interviews that newly employed staff are taken through an orientation process during which IPC training is also done. In TH however, a midwife who had been working on the ward for over two years informed us that she had not received any IPC training. Apart from that, other staff, including the orderlies in both hospitals informed us that they had received IPC training within the past year. There was however no available structure for refresher trainings for staff.

On the maternity wards in both hospitals, there were posters of clinical protocols, guidelines for emergencies, and pictures portraying the importance of hand hygiene, demonstrating hand washing with soap and water, or how to use the alcohol hand rub (also called sanitizer), etc.

Discussions on IPC were sometimes a part of the agenda at monthly unit meetings, where issues related to patient care were discussed and shared to improve the quality of care.

Some nurses described how they implemented knowledge acquired from IPC training and workshops. For example, a nurse described efforts at waste segregation and the use of safety boxes on the wards:

Like our safety box here, we have one in every suite, and we discard the needles appropriately. In addition, we also separate our waste bins. We have bins for infectious waste and general waste. (IDI-2 F, Midwife).

From our observations, however, waste was often not segregated as described. Whereas health providers talked about the use of color-coded bins, a lack of compliance with color codes was observed by the research team several times on the wards. Some health providers explained that the resources including colored bin liners were sometimes not available for use.

Patient education and counseling were perceived to improve attitudes toward hygiene practices. A midwife informed us that patient engagement is best done at the Antenatal Care (ANC) clinics where women receive most of their education during pregnancy. We found that these avenues also enabled healthcare providers to interact with mothers and provide health information. Some of the women we interviewed had been referred from other hospitals for further treatment at TH or SH. Although they attended ANC clinics at other facilities, there were similarities in the information they received at the various hospitals. A mother stated:

I learned about nutrition and how to protect myself from illnesses. When you’re pregnant, there are a lot of factors to consider so you have to watch what you eat (AC1).

Some of the women shared what they could recall from the health talks with nurses during their hospital visits. When asked what they understood by the term puerperal infection, women gave varied explanations such as: “extensive bleeding after birth.” (AC 4), “a sickness you get when you deliver or when you are operated on…” (AC 7). Other women went ahead to describe symptoms such as *weakness and chills, increased temperature, bleeding, headaches*, and *loss of appetite*. A participant shared an experience where she felt her wound was exposed to water, causing an infection. She described that “the wound started smelling, and some water too started coming out from there”.

Due to the approach of sharing information to large groups of women at a time, it appeared there was little opportunity for women to engage actively and seek clarity on the information shared during the educational talks. In addition to the health education by HPs, some women described obtaining information from their peers, family members and community members.

### IPC as a form of self-care for both health providers and women

In describing her motivation for performing hand hygiene, a nurse in TH said:

You have to think about yourself… because if you think about your own health, anything you do, you have to wash your hands (IDI-4 F, Nurse).

Where patients were perceived to be neglecting self-care, some HPs discussed the risks of infection if relevant steps were not taken. During the interviews, a nurse explained that some women sometimes had to be prompted to practice personal hygiene on the ward:

Sometimes, some of them don’t take their bath because they have been operated upon… one time a colleague of mine had to pull one all the way to the washroom… to take her bath, because she started becoming offensive.

A nurse narrated:

I think that for some of them, when they are in labor, they don’t even take their bath until they come here, so their skin is dirty. To set IV (intravenous) lines on them, we do alcohol swabbing, but sometimes you are in a hurry, and you wouldn’t do it very well before you push the needle in. So, I think that one too, infection can occur. (IDI-1 F, Midwife).

Another nurse added that when setting IV lines,

You have to wear gloves and clean the skin surface so that you are assured that you and the patient are protected. (IDI-5 F, Midwife).

A nurse explained that whereas she and her other colleague nurses would usually put on gloves to discard the body fluids of patients, they sometimes observed some women asking their relatives to go and discard their urine for them *‘without gloves’.* She also mentioned that some women preferred to be cleaned and cared for by their relatives rather than the nurses. She then expressed concern about relatives, who remain in the hospital to attend to the needs of women after childbirth, and their risk of exposure to infections, saying: “They are even exposed more than the patients” (IDI-6 F, Nurse).

Some healthcare providers mentioned that they felt vulnerable to acquiring HAIs from patients and mentioned that some of their colleagues had previously acquired infections because of providing care to patients. A nurse indicated that exposure to blood and body fluids during the clamping and cutting of the baby’s cord after delivery exposes one to a risk of infection. Some healthcare providers feared that they might be exposed to infection by touching patients during a clinical examination. A nurse narrated:

Sometimes after delivery, if the client has a tear, you have to suture… and maybe the person has STDS, and you can get it through blood contact.

When you are checking contractions, you have to put your hands on the person and monitor for 10 mins before removing your hands. And also, body fluids… So, if the person has Hepatitis B, you are exposed to that (IDI-6 F, Nurse).

Some women also described some steps taken for self-care to prevent contracting infections. One woman said:

If I am going to use the washroom, I go with my Dettol and everything, to ensure I don’t go and pick up any diseases… When I am done, I wash my hands with soap and water neatly (AC 12).

During the FGDs, women explained that keeping wounds clean and changing wound dressings daily was important. More than half of the women believed that it was important to bathe once or twice daily after childbirth.

### Barriers to IPC compliance

IPC barriers comprised the ‘invisibility’ of HAIs, lack of prioritization of IPC by hospital leaders, lack of goals and sufficient activities for IPC care, discretionary use of protocols, and communication-related challenges.

#### The ‘invisibility’ of HAIs, and variable perceptions about HAIs and HAI risks

When asked about HAIs, most healthcare providers did not have any experiences or encounters to share. They indicated that there were no available documents on HAI on their units that could be referenced. Two healthcare providers said they had ‘no experience’ with HAIs on the wards. A midwife said:

For here, I have not seen any, because here, they don’t keep long here … the maximum they can spend here is maybe 20 hours … because after delivery, after 6–8 hours, they have to move into the ‘lying-in’ ward. (IDI-3 F, Midwife, SH).

Another nurse referred to “the usual ones, usually the cold and stuff. The typical ones like getting pricks”. She added “Some do get pricks; I have been a victim before” (IDI-7 F, Midwife).

About a third of the health providers interviewed mentioned concerns about needle pricks:

… but there was one orderly who had a needle prick and was put on medication. That was just some months ago (IDI-3 F, Midwife, SH).

A nurse explained the possible risks of getting a needle prick if one does not take precautions when setting IV lines and said, “… the patients also have the risk of getting the infection and you have the risk of getting the prick”.

Women in the FGDs perceived themselves to be at risk of infections when healthcare providers attend to them without observing hygiene protocols. A woman said:

If they don’t take the right precaution, such as wearing gloves when putting their hands in your vagina, it can give you infection (AC 10).

Several of the women associated infections with unclean washrooms, unclean beds, lack of hand hygiene among healthcare providers, and lack of personal hygiene among mothers. During the FGDs, two of the women mentioned that puerperal infections may have underlying spiritual causes. A woman mentioned that walking around barefoot can cause an infection.

#### Low prioritization of IPC-related tasks

In TH, the IPC coordinator indicated that his concerns about IPC are only channeled through to the management level by his supervisor if his immediate superior is interested enough in the ideas to communicate them to the next level where management has the authority to act on them. He also mentioned that he did not feel adequately supported to perform his role, for example, his immediate supervisor did not give him the required scheduled time off clinical work to perform IPC monitoring.

From observations, prioritization of tasks was driven by how much attention was given to the task by the more senior health providers, including the Matron- in-charge. Senior nurses delegated some nursing procedures, such as wound dressing, and activities, such as cleaning, to junior nurses, students, or healthcare assistants. Likewise, senior doctors delegated tasks to junior ranks during ward rounds. Delegation of tasks meant staff also decided what would be prioritized, what would be done urgently, and what would be passed on or left for later.

Some tasks were delegated to health assistants or students. These student nurses were expected to know the rationale behind these tasks and have the competency to carry out these tasks or ask for help if needed. However, observations revealed that this was not always the case. Junior nurses commonly described how they had to learn and find things out by themselves. A nurse shared an experience from her early days on the ward:

… nobody oriented us, we just came to the ward and started working. So sometimes I get very confused when they say ‘go here and get me this’. I am learning and finding out things around here by myself.

Aside IPC-related tasks, some procedures are delegated to health assistants or students. This may be associated with complications and infection risks.

A mother narrated:

… after I finished delivering, they had to remove the ‘dirty blood’ or blood clot (placenta); a student showed up and asked Auntie M (midwife) to teach her how to remove it. So, she left it for her, and while removing it, she didn’t remove it all… and some was retained. I kept bleeding heavily afterward. At first, I thought it was because of the delivery. Even after transfusing me, I was still bleeding. And they went to examine me and found that there were retained clots.

#### Lack of resources and sufficient goals for IPC

Health providers in both hospitals expressed the need for more equipment and resources to enable them to do their work more effectively. A doctor mentioned that opportunities to practice IPC were affected by resource limitations:

… for two days, there has been a water issue, but even then, they got water in the barrels so that we could still wash our hands. (MD1)

Another nurse reiterated that the wards rely on water fetched into barrels for handwashing when the tap ceases flowing unpredictably. Speaking of logistic challenges, a manager said:

For example, if you take spirit gel, it’s a little expensive on the market, so we’ve been trying to get our pharmacy to produce it. They did, but we ran into some problems with some machines, which we are trying to solve, so maybe in the next month or two we should be able to get over that.

A few times too, we may run out of a particular color code of lining (for the waste bins). Aside that, we’ve not had too many challenges. It’s all about how well you want to be committed to it.

The aesthetic value of cleaning was emphasized by both healthcare providers and orderlies. Otherwise, no clear goals for IPC were mentioned by participants during the interviews. Orderlies like Alhassan often focused on sensory cues and explained that it was important for the ward to also smell nice. To achieve this, Alhassan described the meticulous process of sprinkling extra bleach on the floor and leaving it for a period to remove any unpleasant smell, then carefully adding nice-smelling detergent to a bucket of water used for mopping the corridors.

However, some HPs mentioned that a clean-looking floor does not necessarily mean there are no germs, as germs cannot be seen with the naked eye. Nevertheless, they agreed that it was important to keep the floors clean and keep the ward smelling nice.

A nurse said:

The place is cleaned but not to the extent that you can’t get an infection. The place has been cleaned in such a way that you can use it, but you must still protect yourself.

Another midwife added:

They can use dirty water and clean the place. You might think the place is clean, but actually, the water used may be infected by other organisms… but physically you see it to be clean… but not necessarily free from organisms. In our system, we use detergents and disinfectants together so that as we are tackling the infections, we are also tackling the dirt as well.

Orderlies also sometimes used their discretion to decide which duties were “part of their job”, as there seemed to be no clear job description, and no clear set of activities for them to perform. HPs sometimes complained that some orderlies also ‘disappeared’ from the wards after early morning cleaning to attend to their personal business. This also happened when the orderlies were sent on casual errands by HPs.

During observations, our attention was drawn to empty alcohol hand-rub dispensers in the unit. It was suggested that a nurse refill these dispensers daily. However, it became evident that the orderlies were expected to refill the dispensers and fill in the gaps for tasks to which no one had been assigned. This was not effective without supervision.

#### Discretionary use of guidelines

According to some heealthcare providers, their hygiene practices were sometimes influenced by what they could see or smell, or what was subject to be inspected or noticed. In preparation for ward rounds, nurses ensured that the ward and working spaces were looking clean and smelling good. Although this was done on most mornings, extra attention was given to this process on the mornings of major ward rounds. They considered this as important, as the matrons could also come round for inspection in preparation for major ward rounds, which were held on specific days, once or twice a week. Nurses felt that preparing ahead for these tasks allowed them to be better positioned to be of service and respond to requests from their supervisors.

We engaged some of the health providers in discussions about the use of protocols and IPC guidelines in their line of work. Some of them indicated that they did not often prioritize the use of guidelines. A midwife said:

Sometimes I read … and you know… because of the busy schedule of the ward, sometimes when you come, you have to take up so that the previous people can go home and go and rest. However, sometimes when I am free, sometimes I take them and read (IDI-1 F, Midwife).

A matron-in-charge, when asked about the antibiotic policy of the hospital, answered:

It is something that is taught in school; it’s not like the hospital should get a policy for you… For any medication, you have to know the patient you are giving it to, whether the drug is expired, and the strength you are giving. Everything is being taught already; it is part of your midwifery or your nursing training. You don’t have to get a hospital policy before you follow that. (IDI-3 F, Midwife).

Another midwife answered:

Antibiotic policy? If there is one, I wouldn’t know. We don’t normally prescribe because we have doctors around.

Speaking about IPC compliance during procedures, a nurse at TH said:

You know… if we are dressing the cord for babies… like 20 babies... I don’t think you expect me to wash my hands after each cord dressing.

A doctor explained that he was not aware of any protocols guiding the insertion or removal of intravenous (IV) lines, but added:

…from what we have learnt, we know that cannulas and catheters should not be left in there for a long time… it could be a source or focus of infection, so it is best to change it after some time.

During interviews and observations, an orderly in TH described a way of cleaning, where ‘high-risk’ spaces are segregated from ‘low-risk’ spaces. He explained that he would, for example, clean the matron’s office with a different mop from what would be used to clean the patients’ bathroom. He indicated that this was his interpretation of what was described in the National IPC policy document, as was explained at the last training session he attended.

A midwife raised concerns expressed about how mops were handled, without following any protocols:

My concern is the mop. The orderlies… they put on gloves and then they use the mop. Maybe they have already touched something infectious with the gloves. Somebody else may touch the mop without gloves and get infected. So, for me, I don’t touch the mop.

We also observed mops being left to dry in open or closed spaces, and even around or in drainage pipes.

In TH, staff would strictly use designated washrooms, which were locked and the key only accessible to staff. They would typically not visit the patients’ washrooms. A midwife in TH described the patients’ washroom as “always dirty”, and associated it with a high risk of infection. She mentioned that she advises the women on admission to take along their disinfectants whenever they use the toilets.

In contrast, the washrooms at SH were maintained well. Describing the patients’ washroom, an HP in SH said:

I won’t say very clean… but it’s clean, because there are different people who come here with different attitudes … Yes, because somebody will go, clean the place or use the place in a nice way but the other person will go and mess up the place for everybody else.

During observations in SH, the bathroom and washroom area often had a clean look. The matron-in-charge attributed it to regular cleaning by the cleaning company to which the cleaning work had been outsourced. This was further supported by a senior manager who said:

Our orderlies were doing fine but then if you look in terms of supervision it became dicey. Is it nurses supervising? Is it administrators? Is it an environmental officer? … if nurses come and they’re concentrating on their work, they wouldn’t go to washrooms to check if they’re clean or not. And the washroom is a very important place for patients. So initially we outsourced the cleaning of the washrooms … and the result was okay. It was good.

#### Communication and information sharing about IPC

Some of the women interviewed reported that health providers failed to communicate with them about their health issues and how to take care of themselves after caesarean sections. A participant shared her experience of how health providers failed to give her information on how to treat her wound after surgery, resulting in wound infection. She narrated that she was disturbed when she was told about the “infection in the wound” and added that the health providers did not tell her the cause of the infection. The woman further explained that although she was worried, she could not gather the courage to make further inquiries about the cause of the infection.

Several women discussed the support of their partners and other relatives, such as mothers, in-laws and grandmothers, in their care. During the FGDs, some women mentioned that they were advised to use alternative or herbal treatments rather than what had been prescribed by health providers.

About half of the women mentioned that they use steam from hot water to help them recover after childbirth: One said:

I sit on hot water, and if there are any herbal medications, I drink them (AC5).

Another woman also mentioned “Dawadawa” (*Parkia biglobosa*- dried seeds of the African locust bean) as an example of a herb that “helps heal birth injuries”. Two women mentioned that they previously boiled and consumed a local herb called “Bagaruwa” (Acacia seeds) to help heal the womb after childbirth.

## Discussion

This qualitative study employed in-depth interviews, focus group discussions and observations of healthcare providers and women in the postnatal period, to explore the factors that influence IPC practices in the maternal wards of two hospitals in Ghana against the backdrop of reducing HAIs.

This study provides evidence that the low IPC compliance in the maternal wards derive from the poor documentation or ‘invisibility’ of HAIs, low prioritization of IPC tasks, lack of IPC goals and resources, discretionary use of guidelines, and communication-related challenges. Whereas hospital leaders described their drive to promote IPC, frontline staff have indicated a need for more support for the provision of resources and an environment conducive to the practice of IPC. Training and education have been described to help improve IPC practices, but training is not structured for HPs, and education for women is not focused on IPC.

Hospital managers in this study were motivated to prevent HAIs, partly to save costs, and to have a good institutional image. Authoritative power (power over) flowed vertically from hospital managers to doctors and nurses at the frontline. At the frontline, healthcare providers also made decisions related to service delivery and IPC compliance based on several factors including personal motivation (power within), and resource availability, which was largely influenced by the managers.

Hygiene behaviour is often perceived to be self-protecting and motivated by the instinct to keep oneself safe from harm. Healthcare providers in this study were influenced by the desire to protect themselves from infections. This is a personal initiative where individuals use the ‘power within’ to make decisions on what actions to take in a given situation [21, 22]. Some studies have also described hygiene behaviours as socially mediated responses to dirt [39, 40]. Healthcare providers in this study made intentional efforts to protect themselves when they felt a particular procedure, process, or contact increased their infection risks.

In this study, some health providers disclosed instances where they waved off hygiene protocols. Similarly, other studies report that health workers consider the processes of hand hygiene and wearing PPEs in between handling patients to be burdensome, with the potential to interfere with their duties and place patients at risk of adverse outcomes [41–43].

Nurse managers exercise the authority to delegate tasks to lower cadres of staff, whom they have ‘power over’. However, a lack of supervision could mean that tasks are poorly performed or not prioritized. As described in other studies, prioritization of decisions may be based on individual judgments about the perceived urgency or importance of a task relative to other tasks [44, 45]. Prioritization has been considered “a precursor to missed care”, as the order in which tasks are completed is based on how highly the tasks are ranked. Moreover, tasks considered outside one’s scope of work are often given a lower priority or left undone [45, 46]. Whereas delegation of tasks may be adopted as a strategy to manage workload, it may negatively affect the quality of care rendered especially when supervision is poor.

Doctors in this study appeared to have a level of power that influences clinical decision-making. Nurse managers also have a critical role on the wards, and their authority and creativity have significant impacts on procedures and tasks [47]. Other studies have reported that health managers derive power from their positions within the hierarchical structure and from resource control [18]. According to Gilson and Lehman [19] high-level actors exercise authoritative power from their hierarchical positions or control over finances. This includes power over IPC budgets and resource allocation.

For frontline healthcare providers, working with inadequate resources causes significant challenges in quality improvement. It is indeed frustrating to work without sufficient water, a basic requirement for a hospital, and more so, on a maternity ward. However, lower-level actors also exercise discretionary power to render services and implement guidelines as they deem fit. To reduce any form of social conflict and promote relationships that can be leveraged to improve IPC, it is important to negotiate and find common grounds and build collective strength [21, 22] to work toward achieving IPC goals.

Healthcare providers who assumed roles as IPC champions did not always receive adequate support from their superiors who in this context hold authoritative power. The exercise of power is used detrimentally when managers do not support other staff to perform in their respective roles, which may undermine the achievement of objectives [19]. Other studies have shown that health providers feel demotivated when supportive behaviours are not offered by hospital managers [48, 49]. The provision of the necessary work equipment, and favourable work environments is essential for quality service delivery [9]. The lack of managerial support for the provision of hygiene equipment makes it of further interest to investigate how managers and relevant power holders can be motivated to pay more heed to this. Studies have shown that although doctors and managers are aware of the importance of IPC, for many, it is not their highest priority [50]. In this study, managers also indicated a need for more support from policy makers, who may not necessarily access the hospital facilities, and may therefore not feel the impact of the facility-level challenges.

The lack of protocols to mandate the documentation of HAIs and the communication of HAIs to patients may have caused HPs to not appreciate the gravity of HAIs. A lack of data about HAIs has been identified as a barrier to doctors becoming more involved in IPC policy [50]. Power dynamics often involve the distribution of information and decision-making authority [21]. The lack of documented information on HAI incidents on the wards indicate an information asymmetry where healthcare providers may not have access to critical data or knowledge about HAI prevalence and rates. Currently, the consequences of HAIs are not that ‘visible’ to HPs on the wards compared to the consequences of omitting some other tasks or emergencies, which may be associated with incompetence.

The variability in perceptions about HAIs and HAI risks among healthcare providers can be influenced by power structures within the hospitals [18, 51]. When those in positions of authority who have the power to set the agenda and priorities for the wards do not prioritize HAI prevention and control, this may lead to the ‘invisibility’ of HAIs. Veneklasen and Miller [21, 22] emphasize how power can shape decision-making processes and what issues are given attention. Hospital managers need to ensure that IPC and HAI prevention are positioned as central to biomedical practice.

Health providers mentioned poor accessibility of protocols, which is a barrier to accessing information on current evidence-based IPC practices. Beyond that, some health providers indicated relying on their judgment and previous experiences in making decisions, which researchers refer to as ‘mindlines’ [52–54]. In our study, some HPs mentioned how they rarely made reference to policy documents or guidelines, but preferred to rely on what Gabbay and May call ‘mindlines’, defined as ‘collectively reinforced, internalized tacit guidelines, which were informed by brief reading, but mainly by their interactions … and by other sources of largely tacit knowledge that built on their early training and their own and their colleagues’ experience’ [52]. In their approach to cleaning, we observed that cleaners similarly relied on a form of ‘mindlines’. When staff rely on their judgment and past experiences (mindlines), they are, to some extent, exercising their professional autonomy. However, the sources of information that inform these mindlines can be influenced by power structures. Lack of access to the latest evidence-based IPC practices due to poor accessibility of protocols and guidelines may result in variations in care quality and potentially undermine patient safety.

Healthcare providers in our study also referred to considerations about maintaining order on the ward, monitoring women to ensure they comply with ward regulations, and ’curbing’ behaviours that are perceived not to be appropriate. Thus, some HPs followed up on women to ‘enforce’ self-care. When healthcare providers enforce compliance and curtail behaviors, it can potentially undermine patient autonomy. It is important to strike a balance between ensuring patient safety and allowing patients to make informed decisions about their care.

Although there were hygiene concerns about relatives of patients, these issues were not proactively addressed. It has been shown that health-seeking behaviour is influenced by the opinions of peers, family members, and other health system factors [55]. It is therefore important that the focus of care is not only on individuals but also on collectives [56]. Health providers need to consider that patients are members of families who live in households, communities, and so on. As women cannot be disentangled from their networks for the care they need for themselves and their babies after birth, health providers must, in addition to the provision of education at antenatal care visits, target the wider community with relevant biomedical information in a culturally sensitive way to facilitate postnatal care and IPC compliance beyond the hospital.

## Conclusion

This study provided insight into the lived experiences of healthcare providers and women in the postnatal phase in two Ghanaian hospitals and explored factors influencing IPC. These findings point to areas that should be addressed in efforts to improve IPC. Greater emphasis on improving IPC as a form of care can enhance its effectiveness in the reduction of HAIs. Recognition of the connections between task prioritization and quality of care, or the significance of some elements and practices, offers important clues for how IPC compliance can be made easier.

The management team of the hospitals, who are the powerholders, need to make policy guidelines available in easily accessible ways at the ward level where implementation occurs and assist healthcare providers in developing protocols and manuals relevant to the ward context. The responsibility to provide the essential resources for service delivery must be considered a high priority.

Making health providers, who hold discretionary power, aware of the tendency to rely on mindlines [53] as opposed to the use of guidelines may be a relevant step to shape behaviour toward the adoption of evidence-based practices.

Health providers and hospital managers need to create avenues to ensure more interactions with women to promote inclusivity and enhance participation in care.

## Limitations

The participatory nature of the research may have influenced the behaviour of some participants - the Hawthorne effect; however, this effect may have lessened over time as participants became accustomed to the researcher’s presence during observations [57, 58]. Interviews with women were conducted within the hospital, and these participants may be unwilling to be critical of the healthcare providers who are still taking care of their health needs. We took steps to build trusting relationships with them and assured them of confidentiality.

### Electronic supplementary material

Below is the link to the electronic supplementary material.

Supplementary Material 1: **Appendix A**: Semi-structured Interview Guide for Study Participants

## Data Availability

All relevant data generated and analysed during this study are included in this published article. Further anonymized details of interviews are available on request from the corresponding author.
