# Supplementary material for: A qualitative study of infection prevention and control practices in the maternal units of two Ghanaian hospitals
Source: Antimicrob Resist Infect Control. 2023 Nov 13;12:125. doi: 10.1186/s13756-023-01330-z (PMC10641978; doi:10.1186/s13756-023-01330-z)
Supplement: Supplementary file 1 — Supplementary Material 1: Appendix A: Semi-structured Interview Guide for Study Participants [file 13756_2023_1330_MOESM1_ESM.docx]

**Appendix A**

**Question guide for Interviews with Mothers**

| 1 | Tell me about what brought you and/or your baby to the hospital?   - What happened on your arrival at the hospital? - Tell me about your admission experience in the hospital so far (Probe for details) |
| --- | --- |
| 2 | Can you tell me what you know about Puerperal Infections (PIs) after new birth?   - What do you think causes PI in the new mother? - What is the source of your awareness/ information? |
| 3 | How important do you think puerperal infections are in the hospital?   - How do puerperal infections affect patients' safety? - In what ways can PIs be acquired from the hospital? - What other factors within the hospital could have an effect on the occurrence of PIs ? |
| 4 | What are some of the symptoms of PI to look out for after discharge from hospital?   - Have you had a PI during this hospital admission or previously? - Can you describe your experience? |
| 5 | What are some preventive measures for PIs?   - If you have (had) puerperal infection, how would/did you seek treatment or help - Which institution/ setting did you go to seek treatment - Share any other means of treatment you adopted - What personal attitudes may help prevent PIs |
| 6 | Apart from healthcare workers in the hospital, who provides you with advice on what medications to use after child birth?  What/ Who is the source of greatest influence?   - What medicines have you applied or taken after the new birth? - What do you use to clean your birth wounds? |
| 7 | Can you tell me about some things in our cultural setting that may put us at risk of birth infections?   - What are some recommended practices to observe in your home or community after childbirth? - (Probe: *Who influences how these practices are followed through?)* |
| 8 | If you were to receive education on PIs, which approaches / means of communication would you prefer ?   - (Explore common means through which health information is accessed) |

**Question Guide for Interviews with Healthcare providers**

| 1 | Tell me about your work in this unit. We could start with you describing a typical working day. |
| --- | --- |
| 2 | What activities in your daily work do you associate with possible risk of infection? |
| 3 | What are some of the healthcare-associated infections (HAIs) you have encountered in this unit/hospital?   - Probe: Who is affected: Staff? Patients? - Probe: Any known incidences of puerperal sepsis on the ward? |
| 4 | Who is in charge of HAIs (surveillance/prevention) in your unit?  Probe : Does the facility have an IPC team in this hospital? Which professionals make up the IPC team?  (For IPC focal person: How much of your time is dedicated for IPC activities)  (For Managers: How does the management team support the IPC program/IPC initiatives in the unit?) |
| 5 | What could be done to reduce the risk of HAIs?  (Probe: Which areas of infection prevention receive the most attention in your unit?)   - Probe: What is readily available and used? (Gowns, soap, water etc?) |
| 6 | Have you come across the National IPC policy or any hospital standards (i.e., policy or guidelines)?   - Probe: How do you use them in your day-to-day work?   What IPC measures do you know?   - Where did you learn these (Probe: any previous IPC training/ professional training?) - (Probe: any known IPC guidelines?) |
| 7 | Can you tell me about previous training you have had in IPC?   - How was IPC integrated in your professional training?/ |
| 8 | What are the main challenges associated with observing IPC measures in this unit?  Probe: What are some barriers you face in complying with IPC recommendations: |
| 9 | How do you feel about encouraging your colleagues to comply with infection control practices? |
| 10 | What role do you think caregivers/relatives have to play in infection control?  Probe: How are caregivers/ relatives engaged  How are they informed or educated about IPC |
